# Comparison of Three Methods for Diagnosing Trichomoniasis in Female Patients with Sexual Activity Attended at a Hospital in Peru

**DOI:** 10.1155/2023/9528942

**Published:** 2023-11-15

**Authors:** Jaime Rosales-Rimache, Jorge L. Inolopú, Fernando C. Soncco-Llulluy, Leslie Medina-Ciprian

**Affiliations:** ^1^Universidad Privada Norbert Wiener, Vicerrectorado de Investigación, Lima 15046, Peru; ^2^Facultad de Salud Pública y Administración, Universidad Peruana Cayetano Heredia, Lima 15101, Peru; ^3^Carrera de Medicina Humana, Universidad Científica del Sur, Lima, Peru; ^4^Hospital Antonio Skrabonja A., EsSalud, Pisco, Peru

## Abstract

**Background:**

Trichomoniasis is a sexually transmitted infection that mainly affects women. The diagnosis is made by different methods that identify the presence of *Trichomonas vaginalis*; therefore, sensitivity, specificity, and performance are essential to guarantee an adequate diagnosis. Our study is aimed at comparing three methods for diagnosing trichomoniasis in patients treated at a hospital in Peru during the year 2018.

**Methods:**

We designed a cross-sectional study and enrolled women whose vaginal secretion samples were evaluated by direct examination, Papanicolaou staining, and culture in the Diamond medium.

**Results:**

We evaluated 134 women with a mean age of 36.6 ± 12.1 years and a beginning of sexual activity of 18.5 ± 3.0 years. We found leukocyte infiltration, fetid odor, and strawberry cervix in 66.4%, 35.1%, and 18.7%. The prevalence of trichomoniasis by the culture method, direct examination, and Papanicolaou was 32.1, 21.6, and 11.2%. The direct examination's sensitivity, specificity, and diagnostic performance (AUC) were 65.1%, 98.9%, and 82.0%, while for the Pap smear, they were 32.6%, 98.9%, and 65.7%%.

**Conclusion:**

The culture of *T. vaginalis* is the gold standard for diagnosing trichomoniasis; however, direct examination is a fast, specific alternative with good diagnostic performance. The Pap test has low sensitivity and should not be used in settings where the prevalence and risk factors for trichomoniasis are high.

## 1. Introduction

Trichomoniasis is a sexually transmitted infection caused by the flagellate protozoan *Trichomonas vaginalis*, with approximately 142 million new cases annually, and more prevalent among women [1]. Some factors have been associated with trichomoniasis, such as ethnicity, pregnancy, HIV infection, low socioeconomic status, number of sexual partners, sex workers, and advanced age [2, 3]. In Peru, a prevalence of 9.1% in women from marginal coastal areas is registered with factors associated with unprotected sex [4].

The clinical manifestations of trichomoniasis are characterized by the presence of yellow discharge, abnormal vaginal odor, and vulvar itching with signs of macular colpitis (“strawberry cervix”), purulent vaginal discharge, and vaginal and vulvar erythema [5]. However, various studies have reported prevalence of asymptomatic trichomoniasis ranging between approximately 30 and 35% [6–8]. On the other hand, the infection with *T. vaginalis* can lead to complications such as adnexitis, pyosalpingitis, and endometritis [9]. In the case of pregnant women, premature rupture of the membrane, preterm delivery, and low birth weight have been reported [10].

The diagnosis is made mainly in symptomatic people through direct microscopic examination of vaginal secretion samples, where mobile structures of *T. vaginalis* are identified [11]. However, the sensitivity of the direct examination is 66.7%; however, it is considered a low value for a test in the screening of trichomoniasis in risk group [12]. Similarly, the use of the Papanicolaou test allows the diagnosis of trichomoniasis with a sensitivity of 57% in areas where the prevalence is greater than 20% [13]. On the other hand, the culture of *T. vaginalis* has been considered for many years as the gold standard for diagnosing trichomoniasis. It can achieve a sensitivity of 95% in Diamond culture [14]. And in the last two decades, molecular methods such as PCR have been implemented to identify *T. vaginalis*, with sensitivity and specificity of 95 and 98% [15].

Timely diagnosis of trichomoniasis makes it possible to indicate pharmacological treatment schemes and provide medical advice to reduce the risk of future reinfections. In this sense, our research is aimed at comparing three methods for identifying *T. vaginalis* in women treated at the hospital of Peru.

## 2. Materials and Methods

### 2.1. Study Area and Participants

We designed a cross-sectional study and enrolled women treated on an outpatient basis at the Santa María del Socorro Hospital in the department of Ica in Peru. This hospital has a classification level II-1, one of the most important in the region. Between February and March 2018, we evaluated 137 patients in the hospital's cervical cancer prevention and control program. We included women aged 18 years and over who gave their consent to participate in the study. We excluded women with no sexual activity and under antibiotic or antiparasitic treatment for at least 15 days before taking the sample. We also do not consider women who reported vaginal douches or taking local medications in the last 72 hours, sexual intercourse, and abundant bleeding the day before sampling collection.

### 2.2. Techniques and Procedures

We prepared a data collection sheet that allowed obtaining information from the laboratory results and medical report review. We got information on the signs and symptoms (itching, fetid odor, redness of the genitals, and burning on urination) reported by the patients. Colposcopic examination allowed us to identify the presence of bright red dotted lesions (strawberry cervix) and leukocyte infiltrate. We also obtained information on the age of onset of sexual activity.

We sampled the vaginal exudate using two sterile swabs applied to the ends of the vaginal cavity. With one swab, we prepared the smear on the sheet for the PAP method, and we used the other swab to deposit it in a 13 × 100 mm tube with 1 mL of sterile saline solution and used it for direct examination and culture.

Cervical cytology was performed using the Papanicolaou technique (Harris hematoxylin, Orange G and EA65), and following the recommendations of the National Institute of Health of Peru [16]. Two medical specialists in pathology observed the slides microscopically at 40x and 100x. The identification of *T. vaginalis* was according to morphological characteristics such as the presence of an elongated shape (size between 7 and 23 *μ*m), pink in color, and with an oval central nucleus, in addition to being found in the middle of polymorphonuclear infiltrates and epithelial cells.

We performed the direct examination of the vaginal secretion soaked in 1 mL of saline solution and centrifuged for 3 minutes at 2400 rpm. For direct observation, we removed the supernatant from the exudate, placed a drop of the pellet on a slide, covered it with a coverslip, and observed under a microscope at 40x magnification, evaluating rapid back-and-forth motility.

We carry out the cultivation of *T. vaginalis* using Hardy Diagnostics modified Diamond medium. We used a micropipette to aspirate 100 *μ*L of the sampled sediment (during the first hour of obtaining it). We deposited it in a 13 × 100 mm tube containing 3 mL of the modified Diamond medium. We incubated the tubes with the culture medium for 24 to 72 hours at 35°C. After that time, we decanted the supernatant, aspirated 50 *μ*L of the sediment, placed it on a slide, and covered it with a coverslip. We observed the preparations under a microscope at 10x and 40x magnification. We identify *T. vaginalis* according to the presence of parasites with rapid motility from one place to another. We performed the microscopic review daily during the first three days after the inoculum. We considered a negative result when we did not find *T. vaginalis* after the third day of incubation. As a quality control measure of the media, they were previously incubated at 35°C for 24 hours and verified turbidity as an indicator of microbial contamination.

We performed all procedures at the microbiology laboratory of the Santa María del Socorro Hospital.

### 2.3. Statistical Analysis

We describe the general characteristics of the study population, using measures of central tendency (mean) and dispersion (standard deviation) for the variables age and age of initiation of sexual activity. We presented categorical variables such as fetid odor, strawberry-shaped cervix, and leukocyte infiltration in absolute and relative frequencies. We showed the frequency of trichomoniasis as a percentage, with its standard error and 95% confidence interval according to each diagnostic method used. We also used the chi-square test to show significant differences between trichomoniasis and demographic and clinical variables, taking a probability value of less than 0.05 as significant. We compared the results of the direct and Papanicolaou methods versus the gold standard (culture) using the chi-square. The ROC (receiver operating characteristic curve) analysis, with the calculation of AUC (area under the curve), sensitivity, specificity, predictive values, likelihood ratios, false positives, and false negatives, correctly classified and estimated agreement with the kappa coefficient and rate of agreement. We performed statistical analysis using Stata v.17 software (Stata Corp, College Station, TX, USA).

### 2.4. Ethical Aspects

This research was approved on March 20, 2018, by the Universidad Alas Peruanas review committee on RD N°154-2018-EPTM-FCS-UAP. Likewise, we obtained administrative permission from the Santa María del Socorro Hospital, where we carried out the enrollment of patients and the execution of techniques and instruments of the study. We obtained informed consent from the participants, a prior explanation of the objectives, the use of techniques, benefits, risks, and management of information generated in the study.

## 3. Results

We recruited 137 women and excluded one woman with no sexual activity and two women under antibiotic treatment for at least 15 days before sample collection, leaving a final sample of 134 women. The mean age was 36.6 ± 12.1 years (min./max.: 18-73 years) and a mean age of onset of sexual activity was 18.5 ± 3.0 years (min./max.: 12-29 years).  Table 1 shows the clinical manifestations of the study participants. We found that 66.4% of the participants presented leukocyte infiltration, 35.1% had a fetid odor in the vaginal secretion, and 18.7% had a strawberry cervix at the colposcopic evaluation.

**Figure 1:**
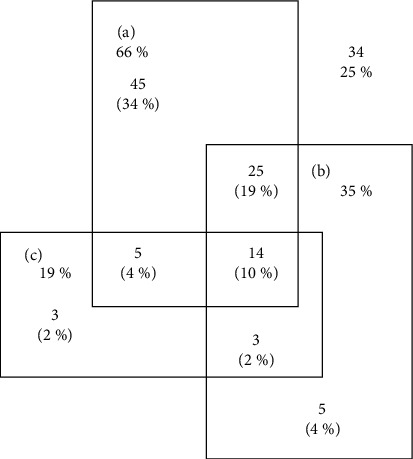
Venn diagram for clinical manifestations of the study population. (a) Leukocyte infiltration, (b) foul odor, and (c) strawberry cervix.

Figure 1 shows that 10% of the women had all three reported clinical manifestations and 25% had none of them.

Table 2 presents the frequency of trichomoniasis, which is greater by the culture method compared to other ones.

Table 3 compares the proportion of trichomoniasis identified by culture according to the characteristics of the participants. We did not find significant differences in the independent variables.

Table 4 presents the results of the comparison of methods for the diagnosis of trichomoniasis. The specificity for the direct examination and Papanicolaou is high and the same (98.9%). However, the sensitivity of the direct examination is greater than that of the Pap smear (65.1% vs. 32.6%). The diagnostic performance of the direct method was higher than that of the Pap smear (AUC 82.0% vs. 65.7%, *p* < 0.001). We found the highest rate of false negatives in the Pap smear.

## 4. Discussion

Our study shows that the cultivation of *T. vaginalis* is the method of choice for the diagnosis of trichomoniasis in women. However, its implementation costs are high, which limits its use as a screening test. On the other hand, the direct examination of vaginal discharge is an easy-to-use alternative in diagnosing trichomoniasis, despite its low sensitivity, but with results closer to culture, compared to the Papanicolaou technique. Culture has better sensitivity than the direct method, mainly because culture positivity requires a minimum of 10^2^*T. vaginalis* trophozoites, while the direct examination requires 10^4^ [17].

It is essential to consider the epidemiological context of trichomoniasis since the sensitivity and specificity of diagnostic tests may vary by prevalence [18]. We have evidenced a frequency of trichomoniasis of 32.1%, taking the culture as a reference. Previous studies in Peru have reported lower frequencies, for example, 10.1% of trichomoniasis in female prisoners, using a combination of direct examination and Pap smear [19], and 7.4% and 5.1% in sex workers and young women, respectively [20]. The lower sexual activity in these last two populations likely generates a lower risk of *T. vaginalis* infection. This scenario is important because it improves the understanding of the behavior of the tests for the diagnosis of trichomoniasis.

Our findings show that the frequency of trichomoniasis varies according to the diagnostic method used. We observed that the frequency increases according to the sensitivity obtained by each test. We have reported a 21.6% frequency of trichomoniasis with the direct examination, whose sensitivity was 65.1%. Previous studies have found 11.0% of trichomoniasis using the direct method and culture (sensitivity 81.8%, specificity 100%) [21] and 12.9% and 6.5% of trichomoniasis with the culture of vaginal discharge [22]. As can be seen, the highest frequency is achieved with the culture, compared to the direct method, also presenting significant differences with the frequency obtained by the culture.

The Papanicolaou test's sensitivity and performance are lower than the direct method. These results may be due to working with fixed and colored smears. These processes can deteriorate the structure, with loss of the flagellum and pyriform morphology of the *T. vaginalis* trophozoite; therefore, it cannot be recognized on microscopic examination. In addition, motility is a characteristic that is not evident in the Pap smear. In this sense, the direct examination performs better than the Pap smear and can even improve with fluorescence microscopy [23].

Trichomoniasis did not have significant differences according to the presence of clinical manifestations such as foul odor and strawberry cervix. This last clinical manifestation shows a high degree of severity of trichomoniasis, considering that they represent microscopic and sharp hemorrhages of the cervix [24]. This alteration occurred in two patients with negative results for trichomoniasis. The small number of participants did not allow for estimating the validity parameters for the evaluated diagnostic tests. Regarding leukocyte infiltrate, this may be of multifactorial origin and may also attribute to infections by *Gardnerella vaginalis* and *Candida albicans* [25]. In our case, we observed the highest frequency in women with trichomoniasis compared to those with a negative result.

Additionally, we reviewed the results of urinary sediment tests in 2017. This result is because, often, the diagnosis of trichomoniasis is given by chance during the observation of the urinary sediment. We found a trichomoniasis frequency of 14.9% (95% CI: 13.1-16.9%) from a total of 1366 urine collections from women with a mean age of 40.8 ± 19.2 years. We found that trichomoniasis was more frequent in women under 30 years of age, in whom the more significant sexual activity was recorded and a greater risk of infection by *T. vaginalis*. Furthermore, we found significant differences (*p* < 0.05) if we compared the frequency obtained by the direct method (21.6%) and the complete urine test (14.9%).

Among the limitations, we did not involve males, even though known that they behave as potential asymptomatic reservoirs of the parasite. Scientific evidence shows that most women are at risk of trichomoniasis and of developing associated complications [26]. We only carried out evaluation of the cultures up to the third day, even though other studies have evaluated the culture for up to 10 days, increasing its sensitivity [27, 28].

An important aspect to consider is that, given the high prevalence found among women, this condition can become chronic without proper treatment and therefore can persist for months and, if left untreated, can increase the risk of HIV infection [29]. *T. vaginalis* could cause an increase in the vaginal load of HIV and therefore increase the risk of transmission of HIV to a sexual partner [30]. On the other hand, the use of molecular techniques such as the rapid detection of *T. vaginalis* antigens and nucleic acid amplification such as PCR and APTIMA TV, among others, is essential for the timely identification of the infection [31].

## 5. Conclusions

The culture of *T. vaginalis* is the method with the highest sensitivity in identifying cases. Direct examination is less sensitive than culture, but it is easier, cheaper, and faster to implement, so its use is ideal for surveillance in risk groups. The Papanicolaou test has low sensitivity, so it is not ideal to use it in diagnostic protocols for trichomoniasis. Finally, it is crucial to consider the evaluation of the sexual partners of women infected with *T. vaginalis*, to avoid the risk of reinfection, considering that men usually behave as asymptomatic carriers.

## Figures and Tables

**Table 1:** Clinical manifestations of the study population.

Clinical manifestations	*N*	%
Leukocyte infiltration		
No	45	33.6
Yes	89	66.4
Foul odor		
No	87	64.9
Yes	47	35.1
Strawberry cervix		
No	109	81.3
Yes	25	18.7

**Table 2:** Frequency of trichomoniasis according to the diagnostic method used.

Method	Frequency (%)	SE	95% CI
Direct examination	21.6	3.6	15.0-29.6
Pap smear	11.2	2.7	6.4-17.8
Culture	32.1	4.0	24.3-40.7

**Table 3:** Comparison of trichomoniasis according to characteristics of the participants.

Characteristics of the participants	Trichomoniasis, *n* (%)		*p* value
	Negative	Positive	
Age (years)	36 (28-45)^a^	32 (23-43)^a^	0.131^b^
Start of sexual activity (years)	19 (17-20)^a^	18 (16-20)^a^	0.098^b^
Leukocyte infiltration			
No	27 (29.7)	18 (41.9)	0.163^c^
Yes	64 (70.3)	25 (58.1)	
Foul odor			
No	59 (64.8)	28 (65.1)	0.975^c^
Yes	32 (65.2)	15 (34.9)	
Strawberry cervix			
No	72 (79.1)	37 (86.1)	0.337^c^
Yes	19 (20.9)	6 (13.9)	

^a^Median and interquartile range. ^b^Mann–Whitney nonparametric test. ^c^Pearson's chi-square test.

**Table 4:** Evaluation of the direct method and Papanicolaou in comparison with culture.

Parameter	95% CI	
	Direct examination	Pap smear
Sensitivity	65.1 (49.1-79.0)	32.6 (19.5-48.7)
Specificity	98.9 (94.0-99.9)	98.9 (93.2-99.9)
Positive predictive value	95.6 (79.8-99.5)	93.3 (66.0-99.7)
Negative predictive value	85.7 (79.9-90.0)	75.6 (66.7-82.8)
Positive likelihood ratio	59.3 (8.3-421.3)^a^	29.6 (4.0-218.1)^a^
Negative likelihood ratio	0.4 (0.2-0.5)^a^	0.7 (0.6-84.0)^a^
False positives	96.6 (80.4-99.8)	6.7 (0.3-34.0)
False negatives	3.4 (0.2-19.6)	24.4 (17.2-33.2)
AUC	82.0 (74.7-89.3)	65.7 (58.6-72.9)
Concordance (kappa)	70.0 (56.8-83.3)	38.0 (22.3-53.7)

^a^Absolute value.

## Data Availability

The data presented in this study are available from the corresponding author on reasonable request.
